# Indications and Outcomes of Intraocular Lens Explantation in a Tertiary Eyecare Center in Hungary between 2006 and 2020

**DOI:** 10.1155/2024/6653621

**Published:** 2024-05-24

**Authors:** Márton Magyar, Nóra Szentmáry, László Ujváry, Gábor László Sándor, Frank Schirra, Zoltán Zsolt Nagy, Gábor Tóth

**Affiliations:** ^1^Department of Ophthalmology, Semmelweis University, Maria Utca 39 1085, Budapest, Hungary; ^2^Dr. Rolf M. Schwiete Center for Limbal Stem Cell and Congenital Aniridia Research, Saarland University, Kirrberger Str. 100 66424, Homburg, Saar, Germany; ^3^Argos Augenzentrum, Faktoreistraße 4 66111, Saarbrücken, Germany

## Abstract

**Purpose:**

Our study aimed to evaluate the indications and outcomes of intraocular lens (IOL) explantation surgeries in a tertiary eyecare center in Hungary.

**Materials and Methods:**

This retrospective study included all IOL explantation surgeries performed between 2006 and 2020 at the Department of Ophthalmology of Semmelweis University, Budapest, Hungary. There were no exclusion criteria for this study. For each patient, the demographics, clinical history, preoperative status, indications for IOL explantation, and operative and postoperative details were reviewed. Primary outcomes included explantation indications and the type of secondary implanted IOL.

**Results:**

A total of 161 eyes from 153 patients were included (96 males; 62.7%); age at the time of the IOL explantation was 65.0 ± 17.4 years. The mean time between primary cataract surgery and IOL explantation was 8.5 ± 7.7 years. In total, 139 (86.3%) PCIOLs and 22 (13.7%) ACIOLs were explanted. The main indications for IOL explantation were dislocation (*n* = 133; 95.7%) and refractive cause (*n* = 2; 1.4%) in the PCIOL group. Among ACIOL explantations, the main reasons were pseudophakic bullous keratopathy (*n* = 14; 63.6%), dislocation (*n* = 4; 18.2%), and refractive cause (*n* = 2; 9.1%). In the PCIOL group, 115 (82.7%) primary IOLs were implanted in the capsular bag, 16 (11.5%) were sulcus fixated, and 8 (5.8%) were scleral fixated. The most frequent ocular comorbidities were previous vitrectomy (*n* = 50, 31.1%), previous ocular trauma (*n* = 45, 28.0%), glaucoma (*n* = 16, 9.9%), pseudoexfoliation syndrome (*n* = 15, 9.3%), and high axial myopia (*n* = 14, 8.7%). The most commonly used secondary IOL implant was the prepupillary iris-claw IOL (*n* = 115, 73.7%), followed by the retropupillary iris-claw IOL (*n* = 32, 20.5%). Uncorrected visual acuity (UCVA) was significantly better following IOL exchange in the entire sample (1.57 ± 0.61 (range: 2.40–0.05) vs. 0.77 ± 0.56 (range: 2.40–0.00); *p* < 0.001). Best-corrected visual acuity (BCVA) was maintained or improved in 80.7% of cases after IOL explantation.

**Conclusions:**

The most common indication for IOL explantation at a tertiary eyecare center in Hungary is IOL dislocation, followed by pseudophakic bullous keratopathy. Prepupillary and retropupillary iris-claw IOL are the most frequently used secondary implants and their use resulted in a significant UCVA improvement following IOL exchange.

## 1. Introduction

Cataract surgery with intraocular lens (IOL) implantation is one of the most commonly performed ocular surgeries worldwide, with outstanding success rates [1]. The surgical techniques and IOL designs are continuously improving. However, unexpected events may occur, rarely those that may require the replacement of the IOL [2]. The incidence of IOL explantation among primary cataract surgeries is estimated to be between 0.59% and 0.77% [3, 4]. Surgical removal and replacement of the IOL can be challenging and are associated with potentially serious complications [5].

Indications for IOL explantation have changed significantly with the introduction of the phacoemulsification technique and foldable posterior chamber IOLs (PCIOL) in cataract surgery over the past few decades. Thirty years ago, during the anterior chamber IOL (ACIOL) era, the most common indications for IOL explantation were pseudophakic bullous keratopathy (PBK), uveitis-glaucoma-hyphema (UGH) syndrome, and cystoid macular edema (CME) [6, 7]. Currently, in the foldable PCIOL age, IOL decentration and dislocation are the most frequent causes (36%). In contrast, the incidence of IOL explantation due to PBK has decreased notably (4%) [8, 9]. Nevertheless, only three up-to-date surveys (from China, Spain, and the USA) [9–11] reporting the indications for IOL explantations are available. Differences in surgical practice and used IOL designs between different continents and countries may cause variations in the need for IOL explantations. For this reason, more up-to-date studies on the causes of IOL explantations are needed [12].

The present study aimed to evaluate the indications and outcomes of IOL explantations performed at a tertiary eyecare center in Hungary.

## 2. Materials and Methods

Our retrospective study was conducted at a tertiary eyecare center in Hungary and included all patients who underwent IOL explantation between January 2006 and December 2020, at the Department of Ophthalmology, Semmelweis University, Budapest, Hungary.

This study was approved by the Regional and Institutional Committee of Science and Research Ethics of Semmelweis University, Hungary (approval no. 95/2022). The study was performed in accordance with the Declaration of Helsinki Guidelines for Human Research.

Clinical data were reviewed for each patient, which included patient demographics (sex, age, and eye laterality), clinical history, comorbidities (ocular and relevant systemic diseases), preoperative and postoperative ophthalmological status, the time interval between primary IOL implantation and explantation, and operative details (concomitant surgeries, cause of explantation, and implanted IOL details). In-the-bag IOL dislocation was defined as dislocation of the IOL with the capsular bag due to instability of the capsular bag or zonular weakness, and out-of-the-bag IOL dislocation was determined as dislocation of the IOL from the damaged capsular bag (e.g., due to capsule tear) into the anterior chamber or vitreous cavity [13]. The included IOL explantations were performed by 23 eye specialists. IOL explantations were performed by enlarging a main incision, bisecting, and removing the IOL. The IOL calculation was based on IOLMaster 500 (Carl Zeiss Meditec, Germany) measurements using the Hoffer Q, SRK/T, Haigis, and Haigis-L formulas. For sulcus fixation, IOL power was reduced by 0.5–1.0 D from the capsular bag power. Scleral-sutured IOLs were fixated ab externo as described by Lewis, wherein sutures are placed from the external surface towards the internal layers of the eye [14].

Uncorrected visual acuity (UCVA) and best-corrected visual acuity (BCVA) data were converted from Snellen chart values to the LogMAR format. Postoperative UCVA and BCVA were analyzed at the 4-month postoperative visit, except for the iris-claw IOL implantations, in which postoperative UCVA and BCVA were analyzed 6 weeks after suture removal.

Statistical analysis was performed using STATISTICA 8.0 (StatSoft Inc., Tulsa, OK, USA). Data were presented as means ± standard deviations (SD). The chi-square test was used to compare the proportions of categorical variables. To compare preoperative and postoperative visual acuity values, we used the nonparametric Wilcoxon test. To compare two treatment groups, we used the Student's *t*-test in the case of normal distribution and the nonparametric Mann–Whitney *U* test for nonnormally distributed variables. A *p* value of <0.05 was considered statistically significant.

## 3. Results

**Figure 1:**
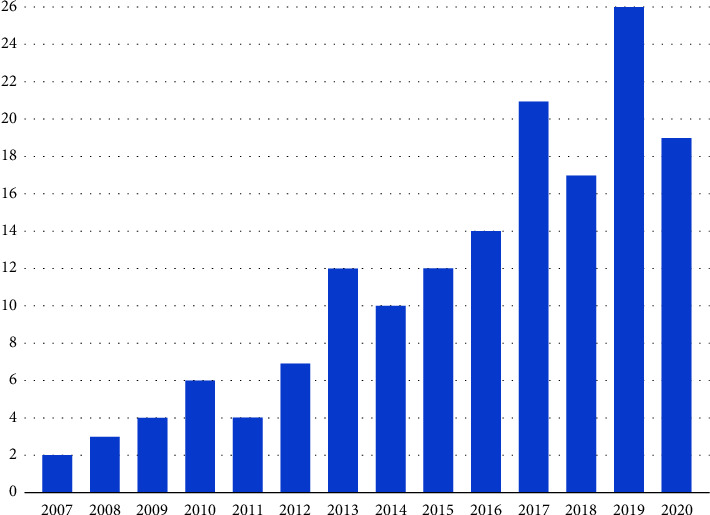
The number of explanted intraocular lenses per year between January 2006 and December 2020 at the Department of Ophthalmology, Semmelweis University, Budapest, Hungary.

During the 15-year study period, 161 IOLs were explanted from 153 patients, consisting of 96 (62.7%) men and 57 (37.3%) women with a mean age of 65.0 ± 17.4 years (range: 2–88 years), among whom, 72 (44.7%) underwent surgery in the right eye and 89 (55.3%) underwent surgery in the left eye. The number of IOL explantation surgeries each year is displayed in Figure 1. Of all subjects with IOL explantation, in total, 80 (49.7%) primary phacoemulsification surgeries with IOL implantation were performed in our department and 81 (50.3%) cases were only referred for IOL explantation to our clinic.

In total, 22 (13.7%) ACIOLs and 139 (86.3%) PCIOLs were explanted. In the PCIOL group, 115 (82.7%) primary IOLs were implanted in the capsular bag, 16 (11.5%) were sulcus fixated, and 8 (5.8%) were scleral fixated. Among all explanted IOLs, 78 (48.4%) were one-piece IOL, 54 (33.5%) were three-piece PCIOL, and 29 (18.0%) were unclassifiable (data were not available regarding the type of IOL).

The main indications for ACIOL explantation were pseudophakic bullous keratopathy (*n* = 14, 63.6%), dislocation (*n* = 4, 18.2%), and refractive causes (*n* = 2, 9.1%). For PCIOL explantation, the main causes included IOL dislocation (*n* = 133, 95.7%) and refractive causes (*n* = 2, 1.4%) (Table 1). The IOL dislocation group consisted of 77 (56.2%) in-the-bag, 50 (36.5%) out-of-the-bag, 6 (4.4%) scleral fixated IOL, and 4 (2.9%) ACIOL dislocation.

The most common ocular comorbidities (Table 2) were previous vitrectomy (*n* = 50; 31.1%), ocular trauma (*n* = 45; 28.0%), glaucoma (*n* = 16; 9.9%), pseudoexfoliation syndrome (*n* = 15; 9.3%), corneal disorders (*n* = 14; 10.5%), high axial myopia (≥26 mm) (*n* = 14; 8.7%), and macular diseases (*n* = 11, 6.8%). The distribution of ocular comorbidities among eyes with in-the-bag, out-of-the-bag dislocation, scleral fixated, and ACIOL explantation group is shown in Table 2. The most common ocular comorbidities were previous ocular trauma (*n* = 43; 33.9%), previous vitrectomy (*n* = 42; 33.1%), and pseudoexfoliation syndrome (*n* = 15; 11.8%) in eyes with in-the-bag dislocation and out-of-the-bag dislocation.

The mean duration between primary cataract surgery and IOL explantation was 8.5 ± 7.7 years in the entire sample (range: 0–33.9 years). The difference between eyes with ACIOL (8.7 ± 7.6 years; range: 0–28.7 years) and PCIOL (13.1 ± 10.8 years; range 0–33.9 years) regarding the mean duration between primary IOL implantation and explantation was not significant (*p*=0.98).

**Figure 2:**
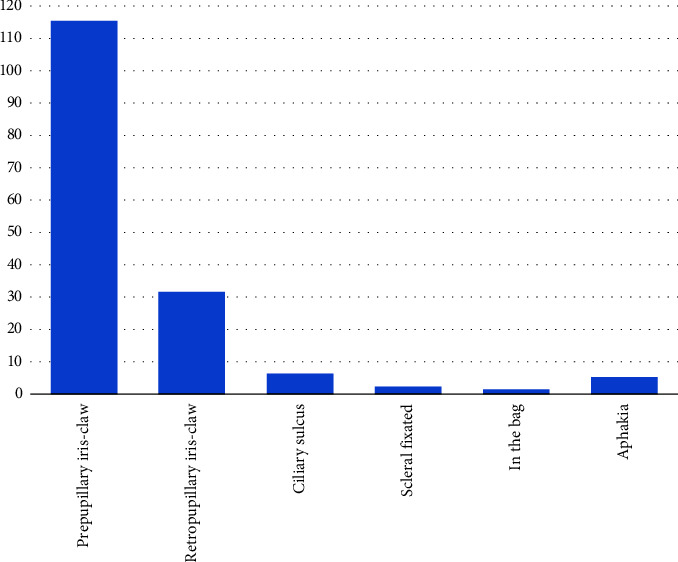
Final location of secondary intraocular lens (IOL) implant after IOL explantation.

Penetrating keratoplasty was concurrently performed in 18 cases (10 combined with ACIOL removal). Anterior vitrectomy was performed simultaneously with IOL explantation in 26 (16.1%) patients and pars plana vitrectomy in 76 (47.2%) eyes. Simultaneous IOL implantation was performed in 131 (81.4%) patients, and 30 (18.6%) eyes were left aphakic after IOL removal. Among the aphakic eyes, 25 (15.5%) received an IOL in a subsequent surgery, and 5 (3.1%) were left aphakic definitively due to poor visual prognosis. Overall, the most frequent secondary IOL implants were the prepupillary (73.7%; *n* = 115) and retropupillary iris-claw IOL implants (20.5%; *n* = 32) (Table 3 and Figure 2).

UCVA before IOL explantation did not differ significantly (*p*=0.96) between the ACIOL group (1.69 ± 0.50 (range: 2.40–0.60)) and the PCIOL group (1.55 ± 0.62 (range: 2.40−0.05)). UCVA was significantly better after IOL exchange in the entire sample (1.57 ± 0.61 (range: 2.40–0.05) vs. 0.77 ± 0.56 (range: 2.40–0.00); *p* < 0.001), in the ACIOL group (1.69 ± 0.50 (range: 2.40–0.60) vs. 1.32 ± 0.52 (range: 2.20–0.50); *p*=0.016), and in the PCIOL group (1.55 ± 0.62 (range: 2.40−0.05) vs. 0.69 ± 0.52 (range: 2.40−0.00); *p* < 0.001). UCVA after IOL explantation and exchange did not differ significantly (*p*=0.46) between the ACIOL group (1.32 ± 0.52 (range: 2.20−0.50)) and the PCIOL group (0.69 ± 0.52 (range: 2.40−0.00)).

BCVA improved in 101 (62.7%) eyes, remained unchanged in 29 (18.0%), and worsened in 31 (19.3%) cases. BCVA did not change significantly after IOL exchange neither in the full sample (0.92 ± 0.72 (range: 2.40–0.00) vs. 0.63 ± 0.56 (range: 2.40–0.00); *p*=0.96) nor in the ACIOL group (1.40 ± 0.70 (range: 2.00–0.20) vs. 0.88 ± 0.42 (range: 1.70–0.00); *p*=0.79) and nor in the PCIOL group (0.87 ± 0.69 (range: 2.40–0.00) vs. 0.57 ± 0.57 (range: 2.40–0.00); *p*=0.63).

Moreover, BCVA did not change significantly after IOL explantation in any indication group (Table 4) (*p* ≥ 0.33).

Twenty-five people (*n* = 25; 15.5%) in the entire sample required topical antiglaucoma agents following the IOL explantation. Significantly more (*p*=0.003) individuals in the ACIOL group (*n* = 8; 36.4%) needed topical antiglaucoma agents compared to those in the PCIOL group (*n* = 17; 12.2%).

## 4. Discussion

There have been many publications on IOL explantation and exchange. Despite advances in surgical techniques and IOL designs, the number of IOL explantations performed annually showed an increasing trend in our study sample. This upward trend is consistent with that reported in Belgium, Spain, the USA, and Turkey [10, 11, 15, 16]. It may be related to the aging of the population and an increasing number of cataract surgeries in developed countries [1, 17].

To the best of our knowledge, our study is the largest single-center study on IOL explantation. Implant-related complications may be addressed by IOL explantation or exchange to improve visual acuity [18].

Our results correlate well with the latest studies. The most common indication for IOL explantation in our study was IOL dislocation (85.1%). This is similar to the rates reported in Spain (56.3% and 81.5%), China (71.4%), and the USA (72.5%) [9, 10, 19].

IOL dislocation itself does not cover a homogenous patient group, therefore we classified further as follows: in-the-bag (56.2%) dislocation and out-of-the-bag (36.5%) dislocation. The predisposing factors and occurrence after primary cataract surgery are shown to be different between in-the-bag dislocation and out-of-the-bag dislocation. In the entire sample, previous vitrectomy, previous ocular trauma, pseudoexfoliation syndrome, and high axial myopia were the most common ocular comorbidities. These are considered the main risk factors for zonular dehiscence, which may explain the high rate of overall IOL dislocation [20, 21]. Previous vitrectomy and previous ocular trauma appear to be a key factor in all groups. According to the current literature, pseudoexfoliation syndrome, high axial myopia, and uveitis were characteristic of in-the-bag dislocation in our sample [13].

The predisposing factors for out-of-the-bag dislocation vary greatly among published studies: complicated primary cataract surgery, previous ocular trauma, previous vitrectomy, mature cataract, pseudoexfoliation, and retinitis pigmentosa, and neodymium-doped yttrium aluminium garnet (Nd: YAG) laser may induce IOL dislocation. Conforming to that, besides glaucoma, previous ocular trauma and vitrectomy were the most common ocular comorbidities in eyes with out-of-the-bag dislocation in our sample.

Patients with Marfan syndrome are prone to developing crystalline lens or IOL luxation, as they may have zonular weakness and loss of capsular support. For this reason, implantation of scleral fixated IOL is the preferred practice in our department following IOL dislocation in patients with Marfan syndrome. [22].

The proportion of ACIOL explantations (13.7%) and IOL explantations performed with penetrating keratoplasty due to PBK (11.2%) were similar in the full sample, because the exchange of ACIOLs is a common procedure in PBK, as the exchange of PCIOL is basically not needed in this disorder [23]. The prevalence of PBK in our study was similar to that reported in Belgium (8.0%) [15], the USA (11.5%) [19] and Spain (12.0%) [10] but higher than that reported in China (4.1%) [9]. A few decades ago, in the ACIOL age, PBK was reported to be the most common cause of IOL explantation in the USA (68.4%) [7]. This change may be explained by advances in IOL technologies, particularly, the introduction of foldable PCIOLs, and small corneal incisions with phacoemulsification. These advances have decreased the use of the ACIOLs over the last few decades [24, 25]. In our sample, 18 penetrating keratoplasties were performed in 10 cases combined with ACIOL removal.

Refractive surprise was the third most frequent indication for IOL explantation, accounting for 1.9% of our cases. As the measurement procedures and calculation formulas evolve, problems related to incorrect IOL calculations should decrease. However, recent studies have reported a higher explantation rate due to refractive surprise, varying from 6% to 18.4% [10, 12, 15, 26, 27]. With careful IOL calculation using adequate modern formulas, the refractive error in 79–95% of patients remains within 0.5 to 1.0 diopter [28]. The lower rate of IOL exchange due to refractive surprise (2.5% in the whole sample, 1.4% in the PCIOL group, and 9.1% in the ACIOL group) in our department may be explained by accurate IOL power calculations and the availability of the excimer laser, which is the treatment of choice following incorrectly calculated IOL and the development of refractive surprise. According to our experience, laser refractive surgery has a significantly lower intraoperative and postoperative risk than IOL replacement [29, 30].

IOL opacification and calcification as the reasons for explantation varied the greatest among available studies, from 1.4% to 31.0% overall contribution [3, 10, 15, 16]. IOL opacification is related to the use of acrylic hydrophilic IOLs. It is now known that the problem is not due to the hydrophilic material itself, but rather a fault in the manufacturing process [3, 31]. The choice of IOL may also vary depending on the country and institution. Manufacturing of the IOLs most frequently affected by opacification (CIBA Vision, MemoryLens U940A and Bausch and Lomb, Hydroview H60M) was ceased in 2000 and 2001 [30]. Considering that this problem was related to a specific type of IOL, the study results may be affected if the problematic type of IOL was implanted in a large number of patients, in a given area [3, 5, 9, 15, 16]. In our study sample, we were unable to demonstrate cases of IOL explantation due to opacification. This refers to a decrease in IOL opacification recently, compared to its frequency in the past.

Multifocal adaptation failure or multifocal IOL intolerance is emerging as a notable indication for IOL explantation. Commonly reported complaints following multifocal IOL implantation include dysphotopsia, halos, glare, and blurred vision at far, intermediate, or near distances [16]. Some studies have reported patient dissatisfaction with multifocal IOLs as a common reason for explantation (6.2–18.3%) [3, 11, 16]. On the contrary, other studies, including those from Belgium [15], the Czech Republic [31], and Spain [10], along with this study, reported no cases of explantation due to multifocal IOL intolerance. This may be because our department is a public hospital where the implantation of multifocal IOLs is rare. However, with the increasing popularity of multifocal IOLs, a growing number of dissatisfied patients are expected to appear, even in public departments.

We did not find any cases of UGH syndrome in our sample. Previously, UGH syndrome was attributed to ACIOLs [6, 7], while today it is thought to be a complication of misplaced three-piece or one-piece IOL implanted in the sulcus [11]. In 2009, a White Paper by the American Society of Cataract and Refractive Surgery and the European Society of Cataract and Refractive Surgery suggested avoiding this practice, as it was associated with high complication risks [32]. In recent literature, UGH syndrome as an indication for IOL explantation varies from 0% to 11.9% [4, 9, 11, 26, 27]. In contrast, 30 years ago, it was one of the leading causes of IOL explantation [6, 7]. The incidence of UGH syndrome may differ according to the different practices at specific institutions. In our clinic, sulcus placement of one-piece IOL is not advised at all. Therefore, we did not identify any explantation cases due to UGH syndrome.

There are different surgical techniques for the removal of the IOLs. Enlarging the corneal incision is a simple and relatively quick technique, although there may be surgically induced astigmatism. Cutting the IOLs (bisecting and trisecting) inside the eye and removing the parts increase the risk of manipulation-caused complications such as endothelial, capsular, iris, and angular structure damage. The twist-and-out technique involves refolding the IOL in the anterior chamber, while the grasp, pull, and refold methods refold the IOL inside the corneal main incision [16, 33].

In our study, the most commonly used secondary IOL implant was the prepupillary iris-claw IOL (73.7%), followed by the retropupillary iris-claw IOL (20.5%). In line with our findings, iris-claw IOLs were the most commonly implanted secondary IOL in Spain (63.8%) [10], but in that study, retropupillary iris-claw IOL was used in all their patients. In contrast, the most frequently used secondary implants in China were the scleral fixated IOL (73.5%) [9]. Scleral fixated IOLs [34] are rarely used in our institution as those were not available for use over several years. During that time period, iris-claw IOLs gained high popularity following their introduction in Hungary.

Interestingly, in the USA, iris-claw IOLs are off-label for aphakia correction. Thus, angle-supported ACIOL in the absence of capsular support is still preferred in the USA [35]. In our clinic, the first choice for secondary IOL implantation is always the capsular bag. In cases where the capsular bag is injured, placement of the IOL in the ciliary sulcus is the second option. Moreover, iris-claw IOLs are preferred in the absence of capsular support. The choice of prepupillary or retropupillary implantation of the iris-claw IOL depends on the surgeon's practice and the patient's eye condition. However, several studies have shown that there is no significant difference in visual outcomes or surgical complications between prepupillary and retropupillary placement of the iris-claw IOL [36, 37].

Most studies have reported significant visual improvement after IOL exchange [4, 8, 10, 12, 26, 27], although postoperative visual acuity depends on the type of explanted IOL and other ocular comorbidities. Our results also showed a significant improvement in UCVA. Besides, BCVA clearly improved in 62.7% of our patients and decreased in only 19.3%.

There are some study limitations. This study used a retrospective design and involved only a single center. Our department is a tertiary eyecare center, and half of the patients were referred to our clinic only for IOL explanation or exchange. For this reason, we could not examine the complete course of these cases or estimate the ratio between the number of IOL explantations and primary cataract surgeries. In this study, data were collected over an extended period, during which it is presumed that techniques and equipment for cataract surgery underwent developments which were not assessed. We collected data from surgeries where IOL explantation was performed, without including surgeries with IOL repositioning. In addition, due to incomplete documentation, intraocular pressure and endothelial cell density could not be analyzed.

## 5. Conclusions

Dislocation and pseudophakic bullous keratopathy were the most frequent indications for IOL explantation and exchange in our department in Hungary. Prepupillary iris-claw IOL was most commonly used for simultaneous secondary IOL implantation and aphakia correction. Consistent with other surveys, we found an increasing trend in the incidence of IOL explantation.

## Figures and Tables

**Table 1:** Indications for intraocular lens explantation (*n* = 161).

Indications	*N* (%)		
	Total	ACIOL explantation	PCIOL explantation
IOL dislocation	137 (85.1)	4 (18.2)	133 (95.7)
Pseudophakic bullous keratopathy	15 (9.3)	15 (68.2)	0 (0)
Refractive surprise	3 (1.9)	1 (4.5)	2 (1.4)
Endophthalmitis	1 (0.6)	0 (0)	1 (0.7)
Unclassifiable	5 (3.1)	2 (9.1)	3 (2.2)
Total	161 (100)	22 (13.7)	139 (86.3)

IOL = intraocular lens; ACIOL = anterior chamber IOL; PCIOL = posterior chamber IOL.

**Table 2:** Ocular comorbidities of patients who underwent intraocular lens explantation.

Ocular comorbidities	Full sample *N* (%)	ACIOL explantation *N* (%)	PCIOL explantation					
			PCIOL dislocation			Refractive surprise *N* (%)	Endophthalmitis *N* (%)	Unknown *N* (%)
			In-the-bag dislocation *N* (%)	Out-of-the-bag dislocation N (%)	Scleral fixated IOL explantation *N* (%)			
Previous vitrectomy	50 (31.1)	5 (22.7)	29 (37.7)	13 (26.0)	1 (16.7)	0 (0)	1 (100)	2 (66.7)
Previous ocular trauma	45 (28.0)	4 (18.2)	29 (37.7)	14 (28.0)	2 (33.3)	0 (0)	0 (0)	0 (0)
Corneal scar, dystrophy, or keratoconus	17 (10.5)	12 (54.5)	2 (2.6)	4 (8.0)	0 (0)	0 (0)	0 (0)	2 (66.7)
Glaucoma	16 (9.9)	2 (9.1)	8 (10.4)	7 (14.0)	0 (0)	0 (0)	0 (0)	1 (33.3)
Pseudoexfoliation syndrome	15 (9.3)	0 (0)	15 (19.5)	0 (0)	0 (0)	0 (0)	0 (0)	0 (0)
High axial myopia (≥26 mm)	14 (8.7)	0 (0)	6 (7.8)	2 (4.0)	0 (0)	0 (0)	0 (0)	0 (0)
Macular diseases	11 (6.8)	2 (9.1)	5 (6.5)	3 (6.0)	0 (0)	0 (0)	0 (0)	0 (0)
Uveitis	8 (5.0)	0 (0)	5 (6.5)	1 (2.0)	0 (0)	0 (0)	0 (0)	1 (33.3)
Marfan syndrome	7 (4.3)	0 (0)	1 (1.3)	3 (6.0)	4 (66.7)	0 (0)	0 (0)	0 (0)
Mature cataract	4 (2.5)	1 (4.5)	1 (1.3)	2 (4.0)	0 (0)	0 (0)	0 (0)	0 (0)
Persistent hyperplastic primary vitreous	1 (0.6)	0 (0)	1 (1.3)	0 (0)	0 (0)	0 (0)	0 (0)	0 (0)
Retinopathy of prematurity	1 (0.6)	0 (0)	1 (1.3)	0 (0)	0 (0)	0 (0)	0 (0)	0 (0)
Microphthalmos	1 (0.6)	1 (4.5)	0 (0)	0 (0)	0 (0)	0 (0)	0 (0)	0 (0)

IOL = intraocular lens; ACIOL = anterior chamber IOL; PCIOL = posterior chamber IOL.

**Table 3:** Final location of intraocular lens (IOL) after IOL exchange.

Indications	*N* (%)	Final location of IOL after IOL explantation and exchange, *N* (%)					
		Prepupillary iris-claw IOL	Retropupillary iris-claw IOL	Ciliary sulcus	Scleral fixated	In-the-bag dislocation	Aphakic
IOL dislocation	137 (85.1)	97 (60.2)	32 (19.9)	3 (1.9)	1 (0.6)	0 (0)	4 (2.5)
Pseudophakic bullous keratopathy	15 (9.3)	14 (8.7)	0 (0)	1 (0.6)	0 (0)	0 (0)	0 (0)
Refractive surprise	3 (1.9)	0 (0)	0 (0)	2 (3.7)	0 (0)	1 (0.6)	0 (0)
Endophthalmitis	1 (0.6)	0 (0)	0 (0)	0 (0)	0 (0)	0 (0)	1 (0.6)
Unclassifiable	5 (3.1)	4 (2.5)	0 (0)	0 (0)	1 (0.6)	0 (0)	0 (0)
Total	161 (100)	115 (71.4)	32 (19.9)	6 (3.7)	2 (1.2)	1 (0.6)	5 (3.1)

**Table 4:** Best-corrected visual acuity before and after intraocular lens explantations.

Indications	Before (LogMAR)	After (LogMAR)	*P* value
IOL dislocation	0.86 ± 0.68 (2.40−0.00)	0.57 ± 0.56 (2.40−0.00)	0.96
Pseudophakic bullous keratopathy	1.68 ± 0.57 (2.00−0.50)	0.99 ± 0.39 (1.70−0.40)	0.92
Refractive surprise	0.17 ± 0.06 (0.20−0.10)	0.05 ± 0.09 (0.15−0.00)	0.33
Endophthalmitis	2.30	0.80	sss
Unclassifiable	0.74 ± 0.50 (1.40−0.20)	0.82 ± 0.41 (1.40−0.30)	0.96
Total	0.92 ± 0.72 (2.30−0.10)	0.63 ± 0.56 (1.70−0.00)	0.71

Data are presented as mean ± SD (minimum-maximum). IOL = intraocular lens; sss = small sample size.

## Data Availability

The data used to support the findings of this study are included within the article.
